# Cascade Responses of Microbial Communities To Alcohols and Organic Acids in a Marine Microcosm Experiment

**DOI:** 10.1007/s00284-025-04581-8

**Published:** 2025-11-13

**Authors:** Ewelina Blanka Grad, Knut Rudi, Julie Martin, Inga Leena Angell, Jenny Helene Mary Storvik

**Affiliations:** Norwegian University of Life Sciences , Ås, Norway

## Abstract

Alcohols and organic acids are major energy-rich components of sewage, and marine environments are significant recipients of these pollutants. However, their effects on marine microbial communities remain poorly understood. Recent theoretical models suggest that high energy inputs can disrupt microbial networks that have evolved under energy-limited conditions. The aim of our study was to determine the community-level effects of two alcohols (ethanol and methanol) and two organic acids (acetate and succinate) using a marine microcosm time-series experiment. We tested three sediment types: one impacted by sewage, one by aquaculture, and one unimpacted. The tested concentrations were 0.05% and 0.5%. At 0.5%, we identified 469 sequence variants (determined from 16 S rRNA gene sequencing) with more than a twofold increase during the experiment, grouped into two enrichment cascades. At 0.05%, 576 sequence variants responded, forming three cascades. Late-appearing sequence variants showed greater inferred functional diversity than early responders. The stronger effects observed at lower concentrations support the theory that energy conservation drives microbial diversification, indicating that even low-level pollution can cause significant ecological changes. These findings highlight the importance of considering microbial community responses in environmental impact assessments of organic pollution in marine ecosystems.

**Supplementary Information:**

The online version contains supplementary material available at 10.1007/s00284-025-04581-8.

## Introduction

Acetate is the main volatile organic component in raw untreated sewage [1], while methanol is the most widely used organic compound for nitrogen removal [2–6]. A range of other carbon sources are also used in wastewater treatment, including ethanol, acetic acid, and succinic acid [7–9]. The general respiratory processes connected to alcohol and organic acid metabolism are relatively well established [10], while we lack knowledge about community effects in marine environments [11].

Marine environments are the primary recipients of sewage in coastal regions. Recent efforts have focused on discharging waste into the aphotic zone, where nutrient dispersion can help prevent eutrophication from excessive algal growth and minimize the impact of human pathogens on recreational use [12]. The impact on the seafloor, however, remains largely unknown.

The seafloor is generally very energy poor [13], with sewage representing a major source of energy rich organic material to the seafloor in coastal regions [14]. A recent theoretical model suggests that division of labor across microbial taxa is energetically favorable under energy poor conditions [15]. High energy input from sewage could therefore disrupt microbial communities selected by the naturally low energy input at the seafloor [16, 17].

In addition to energy level is energy accessibility also important for the impact of alcohols and organic acids on microbial communities. Organic compounds such as succinic acid, which are directly integrated into central metabolism, are generally considered highly accessible [18]. Methanol, on the other hand, is considered to have low metabolic accessibility due to high metabolic cost in creating carbon-carbon bonds [19]. Methanol is also quite stable in the sea, with a lifetime of approximately one week [20], while ethanol which already contains carbon-carbon bonds shows a much shorter half-life in marine environments, being in the range of hours rather than days [21]. The higher oxidative state of acetic acid as compared to methanol and ethanol results in lower energy availability, thereby limiting microbial growth [22].

It has been estimated that methanol conversion to carbon dioxide in nitrogen removal accounts for about 20% of greenhouse gas emissions in the United States from waste-water treatment [23]. This estimate assumes that methanol is entirely converted to carbon dioxide [24]. Under anoxic conditions: however, an unknown fraction of methanol will be converted to methane [25], or potentially other components. Given that the climate impact of methane is 30- to 80-fold higher than that of carbon dioxide [26], methane generation from methanol could have a substantial climate effect.

The objective of this study was to assess the community level impact of alcohols and organic acids on both sediment and seawater microbiota in a microcosm experimental setting, with the aim to unveil potential community level cascade effects and functional diversification.

**Fig. 1:**
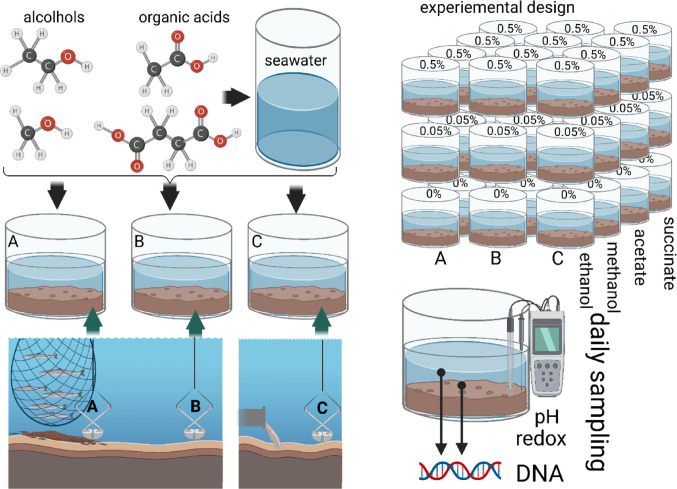
Outline of the setup for the microcosm experiment. Ethanol methanol, acetic acid and succinic acid were separately added at 0.5 and 0.05% in autoclaved seawater to different sediment types; A - Tromsø sea-farm impacted, B - Tromsø non-impacted reference, and C – Oslo fjord sewage impacted. The experiments were conducted at 20 °C in the dark. In total for the microcosm experiment, we evaluated 36 different conditions. For each condition, we analyzed 7 to 10 timepoints, totaling more than 300 analyzed samples

An overview of the experimental design and analytical approaches is presented in Fig. 1.

## Materials and Methods

### Experimental Design

We conducted microcosm simulations using sediments collected close to and distant from a fish farm in Tromsø, Norway (68.83071⁰N 16.03895⁰E), representing a sediment sample impacted by aquaculture, and an unimpacted region from the northern part of Norway. The sampling was conducted in conjunction with governmentally enforced investigations of the aquaculture facilities in December 2022. We also collected sediments from the Oslo fjord (59.59778⁰N 10.65455⁰E), representing the most polluted fjord in Norway, with a long history of sewage pollution [27]. All samples were shipped on ice and stored at 4 °C in the dark prior to usage. The sediments were used within 14 days of sampling.

Ethanol, methanol, acetic acid and succinic acid were separately added at 0.5 and 0.05% volume concentration in 400 ml autoclaved seawater from the Oslo fjord (collected December, 2022). The two concentrations were chosen to reflect DOC levels expected in raw and treated sewage, respectively. The water was subsequently mixed with three different sediment types (100 g, each). From the Oslo fjord, sediment samples were collected in close proximity to a sewer overflow, while in Tromsø, sediment samples were collected both at a fish farm and in a distant region as a reference sample. At the time of sampling for the Oslo fjord sample, the redox potential was − 81 mV, and the pH was 7.7. For the sample collected at a fish farm in Tromsø, the redox potential was 40 mV, and the pH was 7.7. The Tromsø reference sample showed a redox potential of 206 mV, while we did not obtain pH measurements.

The experiments were conducted at approximately 20 °C, in the dark. The temperature was chosen for convenience, as it represents room temperature. The experimental period of 10 to 12 days was determined based on pilot experiments involving tracking of pH and redox potential.

## Measurements of pH and Redox

The pH and oxidation-reduction potential (redox) were measured 3 to 4 times each week using the Hanna HI98121 pH/ORP/Temperature meter (Hanna Instruments, USA).

## DNA Extraction

Sediment sample, approximately 0.1 g were sampled directly to the tube, while 0.1 ml water samples were pelleted at 3 500×g for 5 min. Both the sediments and the pellets were stored at −20 °C until further processing.

Lysis preparation was performed on ice, mixing samples and controls with BashingBead Buffer in BashingBead Lysis Tubes (Zymo Research, USA). STAR buffer served as a negative control, and ZymoBIOMICS Microbial Community Standard mixed with STAR buffer was the positive control. Cell lysis was achieved using a TissueLyser at 30 Hz for two intervals of 2.5 min. After lysis, samples were centrifuged at 10,000 g for 1 min and stored at 4˚C overnight.

Genomic DNA (gDNA) was then separated and purified using a KingFisher Flex robot (Thermo Fisher Scientific, USA) and the Quick-DNA Fecal/Soil Microbe 96 Magbead Kit (Zymo Research, USA). The lysate was mixed with Mag Binding Buffer and Beads, followed by washing with Pre-Wash and gDNA Wash Buffer. The gDNA was eluted in Elution Buffer. The DNA concentration of a subset of samples was measured using a Qubit dsDNA HS kit (Invitrogen, USA).

## DNA Sequencing

Twenty-three µL of a reaction cocktail consisting of 1x HOT FIREPol^®^ Blend Master Mix Ready to Load, 0.2 µM forward primer, 0.2 µM reverse primer and nuclease-free water was added to each well in a 96-well PCR plate, as well as 2 µL template DNA. The plate was amplified using 2720 Thermal Cycler (Applied Biosystems, USA). The following amplicon PCR program was used: 95℃ for 15 min, then 25 cycles of 95℃ for 30 s, 55℃ for 30 s, and 72℃ for 45 s, and a final elongation step of 72℃ for 7 min. The PCR products were checked on a 1% agarose gel for a duration of 30 min at 80 V.

To purify the amplicon DNA products, 1x AMPure XP beads were employed. Vortexed AMPure XP beads were resuspended with DNA in a PCR plate, and after a 5-minute incubation, a magnetic stand was used to separate the supernatant and pellet, discarding the supernatant thereafter. For bead washing, 80% ethanol was added to each well, incubated for 3 min, and the resulting supernatant was discarded. This washing process was repeated twice, and the beads were allowed to air-dry for 15 min. Once completely dry, nuclease-free water was added to each well and resuspended with the washed beads. After a 2-minute incubation in room temperature, the plate was placed on a magnetic stand, and the supernatant was transferred to a new PCR plate.

For the index PCR, each reaction included 1x FIREPol^®^ Master Mix Ready to Load, 0.2 µM forward primer, 0.2 µM reverse primer, nuclease-free water, and DNA from the purification step. DNA amplification was performed using 2720 Thermal Cycler (Applied Biosystems, USA) at 95 ℃ for 5 min, then 10 cycles of 95 ℃ for 30 s, 55 ℃ for 1 min, 72 ℃ for 45 s, and a final elongation step at 72 ℃ for 7 min. Gel electrophoresis was employed to separate the index PCR products on a 1.5% agarose gel, applying 80 V for a duration of 40 min.

The quantification of DNA concentration for the index PCR products was carried out using a combination of gel electrophoresis and Qubit dsDNA HS kit (Invitrogen, USA). In gel electrophoresis, bands exhibiting comparable intensity were considered to possess equivalent DNA concentrations. Additionally, Qubit was employed to measure the DNA concentration of specific samples. The overall DNA concentrations of all samples were quantified in relation to each other, and the samples were pooled to a library.

The pooled library was purified using 0.8x AMPure XP beads and DNA concentrations were checked using Qubit, employing the same protocol as previously described. The libraries obtained were examined using a 2% gel, applying 80 V for a duration of 50 min, prior to shipment to the Norwegian Sequencing Center (Oslo, Norway) for MiSeq v3 300 bp paired end sequencing.

## Bioinformatics

All bioinformatic analyses were conducted using RStudio versions 4.3.1. De-multiplexing is a process used to assign sequence reads to specific samples based on unique barcodes, and in the current study, Illumina paired-end de-multiplexing of raw sequencing data was performed using the demultiplex() function from the midiv package version 2.2.0 (rdrr.io/github/larssnip/midiv/). Furthermore, to assess the sequence quality, FastQC version 0.12.1 was employed to generate detailed quality reports for the forward (R1) and reverse (R2) reads. Additionally, the reports served as references for determining the appropriate parameters for trimming the 3’ ends. Subsequently, VSEARCH version 2.22.1 [28] was used to perform sequence preprocessing, including quality filtering and trimming of low-quality bases from the 3’ ends, trimming 20 bases off the 3’ end of R1 and 60 bases off the 3’ end of R2 before merging. The minimum read length was set to 200, the maximum error probability was set to *p* = 0.01, the minimum copy number of centroid sequence was set to 2, the UNOISE model parameter was set to 2.UNOISE was used for denoising because of its suitability for high-diversity marine seafloor sediments [29].

In the taxonomic annotation step, the zOTU centroids from VSEARCH, which represents the centroid sequences obtained through sequence clustering, served as the input for taxonomic assignment using the SINTAX algorithm [30], while we used the RDP classifier training set number18 for taxonomic assignment [31]. Finally, functional assignments were done using the Faprotax 1.2.7 database [32], using genus assignments from RDP as query.

Prior to further processing, all samples were normalized to relative values from 0 to 1. zOTUs were defined as enriched if they at one timepoint showed a 2-fold enrichment compared to time 0, for a given experimental condition. To penalize low abundant zOTUs, did we multiplied all the relative values with 1000, and then added one prior to determining the enrichment ratios.

### Statistical Analyses

The testing of the overall associations between zOTUs, and the different DOCs were done using ASCA-ANOVA analyses [33]. We applied k-means clustering to processed microbial data to identify distinct clusters based on their enrichment profiles. The effectiveness of this clustering was validated using the Davies-Bouldin and Calinski-Harabasz indices. The statistical significance of the distribution of the number of enriched zOTUs within each genus was determined using the chi-square test. The distribution of relative amounts of taxonomic groups and curve fitting parameters were tested using the Kruskal-Wallis test. Curve fitting was done using a polynomial regression. We used Matlab R2022b (Mathworks inc, USA) and Minitab 18 (Minitab inc, USA) for the statistical analyses.

## Results

### The Overall Microbiota Composition Unveiled Unique Patterns across Sediment Site and DOC Type

**Fig. 2:**
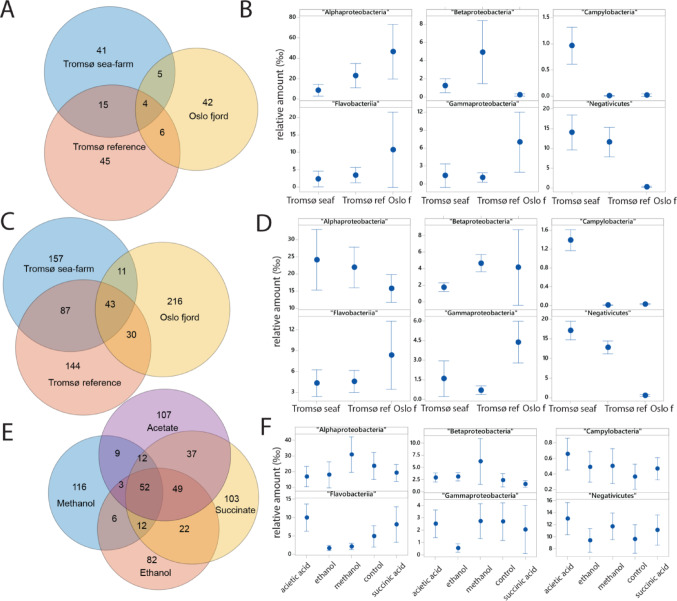
Overall microbiota composition. The overall microbiota composition was determined for the zOTU overlap between sites prior to the enrichment (A), during the enrichment (C), and between the DOCs (E). The mean class level distributionfor the different sites before the enricment (B), during the enrichment (D), and between the different DOCs (F)

We determined the zOTU overlap across the three sites under investigation, prior to the enrichment. These analyses uncovered that about 40 of the zOTUs with levels above 0.1% within a given sample were unique for each of the three different sediment types. Notably, the degree of overlap was more pronounced between the aquaculture impacted and not impacted site, as compared to the Oslo fjord site impacted by sewage (Fig. 2A). In terms of taxonomic differences did *Campylobacteriota* show an overrepresentation for the fish-farm impacted site, *Gammaproteobacteria* showed an overrepresentation for sewage impacted site, while *Betaproteobacteria* displayed an overrepresentation for the non-impacted site. *Negativicutes* was underrepresented for the Oslo fjord (Fig. 2B). During the enrichment, there was an approximate threefold increase in the total number of zOTUs surpassing the 0.1% threshold, with an overrepresentation between Tromsø sea farm impacted and Tromsø non impacted samples (Fig. 2C). Similar geographical patterns were identified during the enrichment for *Campylobacteriota*,* Gammaproteobacteria* and *Negativicutes*, as observed before the enrichment. The *Alphaproteobacteria* and Betaproteobacteria, however, seemed to shift patterns (Fig. 2D).

Regarding the correlation between taxonomic groups and DOCs, approximately 100 unique zOTUs were identified for each of the four compounds (Fig. 2E). Distinct associations between DOCs were not evident from the collective analyses (Fig. 2F).

### Site Showed the Most Pronounced Impact on zOTU Change

For the 0.5% DOC enrichments, 469 zOTUs exhibited a more than 2-fold increase compared to the original sample, whereas the corresponding count for 0.05% DOC was 576.

Site demonstrated the most substantial impact on the change pattern, elucidating 6.34% explained variance for 0.5% DOC and 3.8% for 0.05% DOC (*p* ≤ 0.001 for both), as determined by ASCA-ANOVA. In both cases, the sewage impacted samples also displayed the most distinct pattern (*p* < 0.00005, Kruskal-Wallis). While the type of organic compounds manifested a less pronounced effect at 3.1% for 0.5% and 1.4% for 0.05% using ASCA-ANOVA, it remained statistically significant (*p* < 0.001). Notably, the 0.5% enrichment revealed a more substantial impact, particularly in water phase, accounting for 0.83% of the explained variance (*p* = 0.003, ASCA-ANOVA).

### k-mean Clustering Identified Potential Cascade Effects

**Fig. 3:**
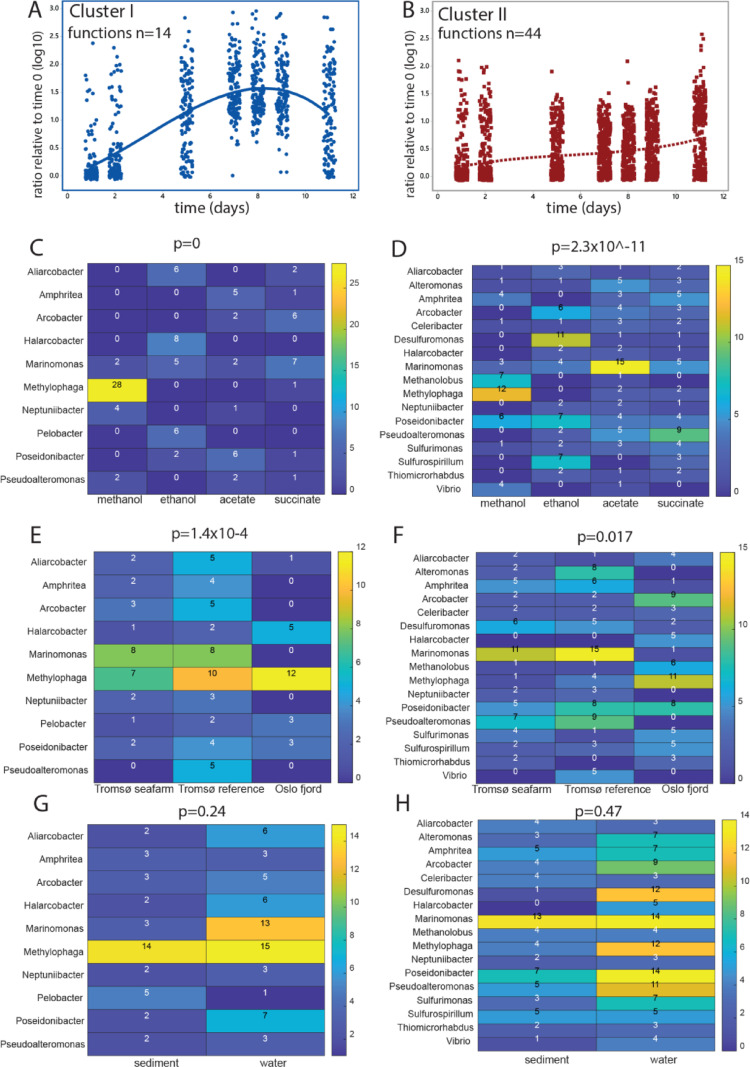
Microbiota response to 0.5% DOC. Panels A and B depict zOTU enrichment distribution for 0.5% Clusters I and II. The plots represent the enrichment distribution for the zOTUs belonging the respective k-mean clusters across the different sampling times. The lines represent the trends as unveiled by polynomial regression. The number of functions represent the collective number of functions for the zOTUs within the respective clusters, as determined by the Faprotax database. Panels C to H represent the number of zOTUs within the respective genera that show a more than 10-fold enrichment at any timepoint, as compared to the original sample for the different categories evaluated. The p-values represent the significance levels for the chi-square test. Panels C and D show the number of zOTUs within genera by DOC type for Clusters I and II. Panels E and F present site-specific variations for Clusters I and II. Panels G and H detail microbiota composition by sample type for Clusters I and II

K-means clustering with Davies-Bouldin and Calinski-Harabasz evaluation identified two clusters of taxa for 0.5% DOC (denoted 0.5% DOC Cluster I and II). The 0.5% DOC Cluster I showed a peak at about 8 days, while 0.5% Cluster II showed a gradual increase throughout the 11 days (Fig. 3A and B). For functionality, as determined by Faprotax there was a clear expansion of functions covered from 15 functions for 0.5% Cluster I to 43 functions covered by 0.5% Cluster II (Suppl Table 1).

At the genus level, the organic compound type showed the most distinct enrichment, with a clear overrepresentation for *Methylophaga* for 0.5% Cluster I from the methanol enrichment (Fig. 3C). For 0.5% Cluster II *Methanolobus* and *Vibrio* also showed an overrepresentation for methanol, in addition to *Desulfuromonas* for ethanol (Fig. 3C). Location also showed distinct enrichments, with *Marinomonas* being associated with the two Tromsø samples (Fig. 3E and F). Water and sediment did not show statistically significant differences with respect to the number of zOTUs enriched for the different genera (Fig. 3G and H).

**Fig. 4:**
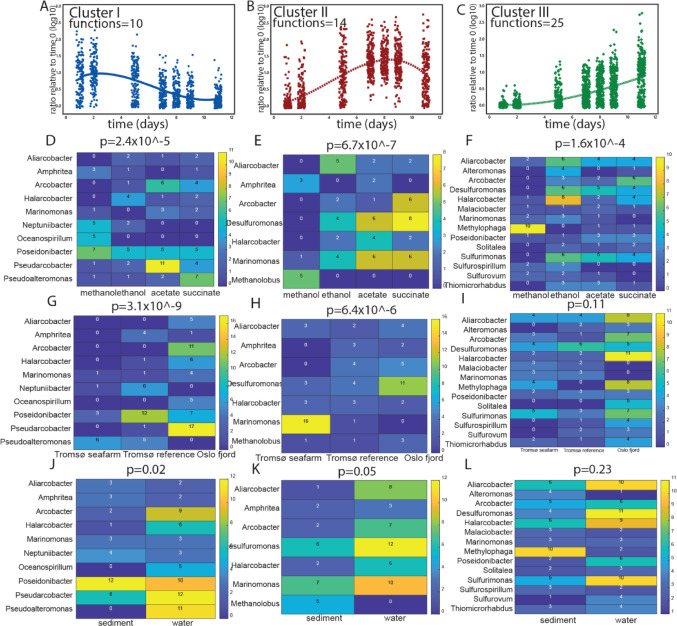
Microbiota response to 0.05% DOC. Panels A to C depict zOTU enrichment distribution for 0.5% Clusters I, II and III. The plots represent the enrichment distribution for the zOTUs belonging the respective k-mean clusters across the different sampling times. The lines represent the trends as unveiled by polynomial regression. The number of functions represent the collective number of functions for the zOTUs within the respective clusters, as determined by the Faprotax database. Panels C to L represent the number of zOTUs within the respective genera that show a more than 10-fold enrichment at any timepoint, as compared to the original sample for the different categories evaluated. The p-values represent the significance levels for the chi-square test. Panels D to F show the number of zOTUs within genera by DOC type for Clusters I, II and III. Panels G to I present site-specific variations for Clusters I, II and III. Panels J to L detail microbiota composition by sample type for Clusters I, II and III

For 0.05% DOC, k-means clustering with Davies-Bouldin and Calinski-Harabasz index evaluation showed three clusters as optimal, 0.05% Cluster I to III. Functionally, nitrate reduction was covered by all clusters. There was an expansion of functions covered, from 10 for 0.05% Cluster I, to 14 in 0.05% Cluster II and 25 in 0.05% Cluster III. Most of the expansion of functions were related to sulfur and metal redox reactions (Suppl. Table 2). 0.05% Cluster I showed a peak after 2 days, while 0.05% Cluster II showed a peak after 8 days. Finally, 0.05% Cluster III showed a steady increase until day 11 (Fig. 4A-C). Both *Methanolobus* and *Methylophaga* showed a clear association with methanol, with *Methanolobus* being associated with 0.05% Cluster II (Fig. 4D), and *Methylophaga* with 0.05% Cluster III (Fig. 4D). The strongest associations detected were towards location for 0.05% Cluster (I) Here, both *Arcobacter* and *Pseudarchobacter* showed a strong association with the Oslo fjord (Fig. 4G). For Cluster II, there was a strong association between *Marinomonas* and Tromsø sea farm impacted sediment (Fig. 4H), while there were no statistically significant associations for Cluster III (Fig. 4I). In contrast to 0.5% organic compounds, we identified statistically significant associations for Cluster I and (II) For Cluster I, *Pseudoalteromonas* showed a strong association with water (Fig. 4J), while *Methanolobus* showed a strong association with sediment for 0.05% Cluster II (Fig. 4K). 0.05% Cluster III did not show statistically significant associations with water or sediment (Fig. 4L).

### Sediment Site Showed Larger Impact on Redox than Organic compound-type

**Fig. 5:**
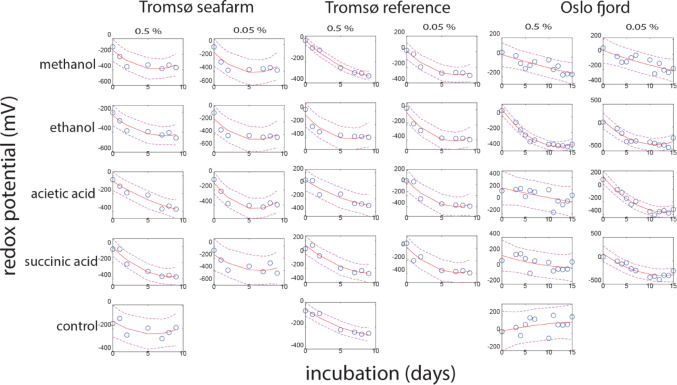
Effect of DOC on redox potential at the interface between water and sediment. For each incubation condition did we perform polynomial curve fitting. The stippled lines represent the 95% confidence interval

According to the redox measurements conducted for the timeseries analyses, all the DOCs caused a noteworthy reduction in the redox potential. The maximum decrease observed was around − 500 mV. Nevertheless, the reaction patterns demonstrated notable variations across the tested conditions, as illustrated by the polynomial regression results shown in Fig. 5.

The estimated parameters in the polynomial models were subjected to a non-parametric Kruskal Wallis test, which revealed that the sediment site had a greater impact on the redox pattern compared to the to the different DOCs. The Tromsø samples displayed more pronounced curvature than the Oslo fjord sample (*p* = 0.007), while the Oslo fjord sample showed a lower linear decrease in the redox potential (*p* = 0.023). Moreover, the Oslo fjord sample had the highest initial redox potential, while the Tromsø sea farm impacted sample had the lowest (*p* < 0.0005). On the other hand, the different organic compounds did not exhibit statistically significant differences in terms of redox (*p* > 0.05, Kruskal Wallis). The curve fitting information is provided in Suppl. Table 3.

### Organic Compound-type Showed Larger Impact on pH than Sediment Site

**Fig. 6:**
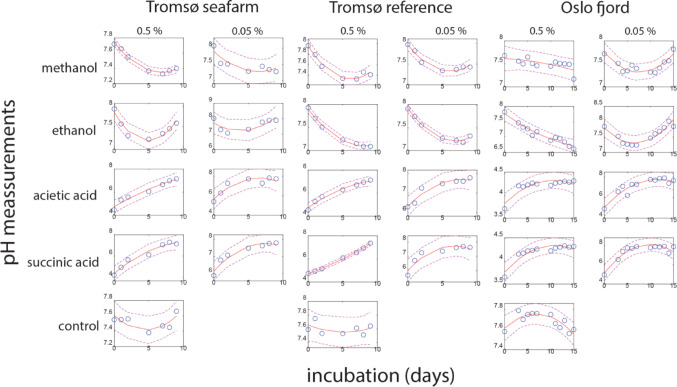
Effect of DOC on pH at the interface between water and sediment. For each incubation condition did we perform polynomial curve fitting. The stippled lines represent the 95% confidence interval

The overall trend observed was that the pH decreased over time for the alcohols, whereas it increased over time for the organic acids. Furthermore, the initial pH for the organic acids was below 5, while for the alcohols it was above 7.5. These patterns have been visualized using polynomial regression in Fig. 6.

The Kruskal Wallis test revealed no significant differences for the sites (*p* > 0.05), whereas the organic compounds exhibited significant differences for all the parameters (*p* < 0.05). In terms of the estimated polynomial parameters, the acids generally displayed a negative curvature, while the alcohols showed a positive curvature (*p* = 0.001). For the linear trends, the acids demonstrated an increase over time, while the alcohols exhibited a decrease (*p* < 0.0005). Finally, for the constant, the acids displayed lower values than the alcohols (*p* < 0.0005). The curve fitting information is provided in Suppl. Table 4.

## Discussion

We observed community-level effects in response to both alcohols and organic acids, with the strongest community effects for the lowest concentrations. These findings align with the recent theoretical models, suggesting that energy-limited systems promote resource sharing and diversity within microbial networks [15]. There was also an apparent increase in functional potential for the community clusters with late appearance, as compared to those with early appearance. This observation also align with energy conservation theory [15], providing a potential mechanistic explanation for chemical diversification of organic carbon in marine environments [34]. Energy conservation through diversification could explain the long-standing controversy of high microbial diversity on the seafloor despite relatively few niches [13, 16, 17]. Therefore, disrupting energy conserving microbial networks at the seafloor could be a general mechanisms for how human activities disrupt marine ecosystems [17].

Methanol induced the most distinct enrichment effect, potentially reflecting the difficulty in metabolism, as methanol lacks carbon-carbon bonds [35]. *Methylophaga* and *Methanolobus* showed a clear association with methanol enrichment. *Methylophaga* has previously been linked to methanol oxidation in marine ecosystems [36], while methanol-utilizing *Methanolobus* has been isolated from a range of marine sediments, being an obligate methanogen performing methylotrophic methanogenesis [37]. This indicates a very strong selective pressure for these genera with methanol. Most likely, *Methylophaga* will be selected under oxic conditions, while *Methanolobus* will be selected under anoxic conditions. The mechanisms for metabolic selection of methyl-based compounds, however, remain poorly understood [25, 38], representing a potential major contributor to the global methane emission [26].

Sediment type showed a pronounced association with both microbiota enrichment profiles and redox potential. The two Tromsø samples exhibited more closely related profiles than those from the Oslo fjord sample. The redox curvature for the Tromsø samples was larger than for the Oslo sewage samples, indicating a more rapid recovery of redox potential for the Tromsø samples as compared to the Oslo fjord sample. This could potentially be attributed to a greater resilience and resistance to perturbations in the Tromsø samples [39, 40]. A potential mechanism for resilience could be provided by *Marinomonas*, which represented the main microbial difference between Oslo fjord and Tromsø samples. Intriguingly, this genus produces an antimicrobial enzyme that oxidizes lysine, releasing hydrogen peroxide as an antimicrobial mechanism [41]. The potentially lower resilience for the Oslo fjord samples could be attributed to the fact that the Oslo fjord has been subjected to pollution for an extended period.

For ethanol and methanol, we observed an initial decrease in pH, which shifted upwards after approximately one week. This process, however, was different for acetate and succinate, where the pH was low initially, while gradually increasing with time. In all cases, the redox potential decreased over time, with the drop being less extensive if the pH was below 5. In all cases, there was a browning of the water when the redox dropped below − 200 mV (empirical observations). Generally, sulfate reduction by organic compounds is a process that consumes hydrogen ions, resulting in an increased pH. However, incomplete reduction of ethanol will lead to acetate formation [42], potentially causing the initial pH decrease observed for the alcohols.

In conclusion, our study further highlights the knowledge gap related to the utilization and fate of anthropogenic alcohols and organic acids in marine ecosystems, showing the potential for community effects and diversification as response to both level and energy accessibility. These effects which could be critical for marine ecosystems, are largely overlooked in seafloor management. We therefore suggest that future research should aim at understanding the fate and consequences of simple DOC in natural marine ecosystems, particularly related to the energy level and metabolic accessibility.

## Supplementary Information

Below is the link to the electronic supplementary material.

Supplementary Material 1

Supplementary Material 2

## Data Availability

The sequences are deposited in the SRA database with the accession number PRJNA1065800.

## References

[1] Narkis N, Henfeld-Furie S (1978) Direct analytical procedure for determination of volatile organic acids in raw municipal wastewater. Water Res 12(7):437–446

[2] Helness H, Melin E, Ulgenes Y, Jarvinen P, Rasmussen V, Odegaard H (2005) High-rate wastewater treatment combining a moving bed biofilm reactor and enhanced particle separation. Water Sci Technol 52(10–11):117–127 16459783

[3] Nyberg U, Aspegren H, Andersson B, la Villadsen JC (1992) Full-Scale application of nitrogen removal with methanol as carbon source. Water Sci Technol 26:1077–1086

[4] Yin F-F, Guo H-F (2022) Influence of additional methanol on both pre- and post-denitrification processes in treating municipal wastewater. Water Sci Technol 85:1434–1443 35290223 10.2166/wst.2022.060

[5] Slade AH, Thorn GJ, Dennis MA (2011) The relationship between BOD:N ratio and wastewater treatability in a nitrogen-fixing wastewater treatment system. Water Sci Technol 63(4):627–632 21330706 10.2166/wst.2011.215

[6] Agency USEP (2013) Wastewater Treatment Fact Sheet:External Carbon Sources for Nitrogen Removal

[7] Strong PJ, McDonald B, Gapes DJ (2011) Enhancing denitrification using a carbon supplement generated from the wet oxidation of waste activated sludge. Bioresour Technol 102(9):5533–5540 21196117 10.1016/j.biortech.2010.12.025

[8] Peng H, Zhang Q, Tan B, Li M, Zhang W, Feng J (2021) A metagenomic view of how different carbon sources enhance the aniline and simultaneous nitrogen removal capacities in the aniline degradation system. Bioresour Technol 335:125277 34004561 10.1016/j.biortech.2021.125277

[9] Brozinčević A, Grgas D, Štefanac T, Habuda-Stanić M, Zelić B, Landeka Dragičević T (2024) Cost reduction in the process of biological denitrification by choosing traditional or alternative carbon sources. Energies 17(15):3660

[10] Fernie AR, Zhang Y, Sweetlove LJ (2018) Passing the Baton: Substrate Channelling in Respiratory Metabolism. Research. ;2018 10.1155/2018/1539325 PMC6750097 31549022

[11] Thommes M, Wang T, Zhao Q, Paschalidis IC, Segrè D (2019) Designing metabolic division of labor in microbial communities. mSystems 4(2). 10.1128/mSystems.00263-18 10.1128/mSystems.00263-18 PMC6456671 30984871

[12] Arnesen V (2001) The pollution and protection of the inner Oslofjord: redefining the goals of wastewater treatment policy in the 20th century. Ambio 30(4–5):282–286 11697263

[13] Orsi WD (2018) Ecology and evolution of seafloor and subseafloor microbial communities. Nat Rev Microbiol 16(11):671–683 29967420 10.1038/s41579-018-0046-8

[14] Tuholske C, Halpern BS, Blasco G, Villasenor JC, Frazier M, Caylor K (2021) Mapping global inputs and impacts from of human sewage in coastal ecosystems. PLoS ONE 16(11):e0258898 34758036 10.1371/journal.pone.0258898 PMC8580218

[15] Seto M, Kondoh M (2023) Microbial redox cycling enhances ecosystem thermodynamic efficiency and productivity. Ecol Lett 26(10):1714–1725 37458207 10.1111/ele.14287

[16] Rudi K, Nilsen T, Pettersen R, Keeley NB, Ray JL, Majaneva S et al (2025) The coastal seafloor microbiota is structured by local selection of cosmopolitan taxa. Environ Microbiol Rep 17(3):e70123 40537448 10.1111/1758-2229.70123 PMC12178751

[17] Nilsen T, Pettersen R, Keeley NB, Ray JL, Majaneva S, Stokkan M et al (2025) Association of microbial networks with the coastal seafloor macrofauna ecological state. Environ Sci Technol 59(15):7517–7529 40214404 10.1021/acs.est.4c12464 PMC12020364

[18] Tretter L, Patocs A, Chinopoulos C (2016) Succinate, an intermediate in metabolism, signal transduction, ROS, hypoxia, and tumorigenesis. Biochimica et biophysica acta (BBA). - Bioenergetics 1857(8):1086–1101 26971832 10.1016/j.bbabio.2016.03.012

[19] Chistoserdova L, Kalyuzhnaya MG, Lidstrom ME (2009) The expanding world of methylotrophic metabolism. Annu Rev Microbiol 63:477–499 19514844 10.1146/annurev.micro.091208.073600 PMC2827926

[20] Dixon JL, Beale R, Nightingale PD (2013) Production of methanol, acetaldehyde, and acetone in the Atlantic Ocean. Geophys Res Lett 40(17):4700–4705

[21] de Bruyn WJ, Clark CD, Senstad M, Toms N, Harrison AW (2020) Biological degradation of ethanol in Southern California coastal seawater. Mar Chem 218:103703

[22] Elefsiniotis P, Wareham DG (2007) Utilization patterns of volatile fatty acids in the denitrification reaction. Enzym Microb Technol 41(1):92–97

[23] Willis JL, Al-Omari A, Bastian R, Brower B, DeBarbadillo C, Murthy S et al (2017) A greenhouse gas source of surprising significance: anthropogenic CO(2) emissions from use of methanol in sewage treatment. Water Sci Technol 75(9–10):1997–2012 28498113 10.2166/wst.2017.033

[24] Dold P, Takács I, Mokhayeri Y, Nichols A, Hinojosa J, Riffat R et al (2008) Denitrification with carbon addition–kinetic considerations. Water Environ Res 80(5):417–427 18605381

[25] Bueno de Mesquita CP, Wu D, Tringe SG (2023) Methyl-Based methanogenesis: an ecological and genomic review. Microbiol Mol Biol Rev. 87(1). 10.1128/mmbr.00024-22 10.1128/mmbr.00024-22 PMC10029344 36692297

[26] The Earth’s Energy Budget (2023) Climate feedbacks and climate sensitivity. Intergovernmental panel on climate C, editor. climate change 2021 – The physical science basis: working group I contribution to the sixth assessment report of the intergovernmental panel on climate change. Cambridge University Press, Cambridge, pp 923–1054. https://www.ipcc.ch/report/ar6/wg1/chapter/chapter-7/

[27] Ruud JT (1968) Introduction to the studies of pollution in the Oslofjord. Helgol Wiss Meeresunters 17(1):455–461

[28] Rognes T, Flouri T, Nichols B, Quince C, Mahe F (2016) VSEARCH: a versatile open source tool for metagenomics. PeerJ 4:e2584 27781170 10.7717/peerj.2584 PMC5075697

[29] Nilsen T, Snipen L-G, Angell IL, Keeley NB, Majaneva S, Pettersen R et al (2024) Swarm and UNOISE outperform DADA2 and Deblur for denoising high-diversity marine seafloor samples. ISME Commun 4(1):ycae071 38873028 10.1093/ismeco/ycae071 PMC11170925

[30] Edgar RC (2016) SINTAX: a simple non-Bayesian taxonomy classifier for 16S and ITS sequences. BioRxiv. :074161

[31] Wang Q, Cole JR (2024) Updated RDP taxonomy and RDP classifier for more accurate taxonomic classification. Microbiol Resour Announc. 13(4):e0106323. 10.1128/mra.01063-23. PMID: 38436268; PMCID: PMC11008197. 10.1128/mra.01063-23 PMC11008197 38436268

[32] Louca S, Parfrey LW, Doebeli M (2016) Decoupling function and taxonomy in the global ocean microbiome. Science 353(6305):1272–1277 27634532 10.1126/science.aaf4507

[33] Smilde AK, Jansen JJ, Hoefsloot HC, Lamers RJ, van der Greef J, Timmerman ME (2005) ANOVA-simultaneous component analysis (ASCA): a new tool for analyzing designed metabolomics data. Bioinformatics 21(13):3043–3048 15890747 10.1093/bioinformatics/bti476

[34] Dittmar T, Lennartz ST, Buck-Wiese H, Hansell DA, Santinelli C, Vanni C et al (2021) Enigmatic persistence of dissolved organic matter in the ocean. Nat Rev Earth Environ 2:570–583

[35] Le T-K, Lee Y-J, Han GH, Yeom S-J (2021) Methanol dehydrogenases as a key biocatalysts for synthetic methylotrophy. Front Bioeng Biotechnol. 9:787791. 10.3389/fbioe.2021.787791. PMID: 35004648; PMCID: PMC8741260. 35004648 10.3389/fbioe.2021.787791 PMC8741260

[36] Neufeld JD, Neufeld JD, Schäfer H, Cox MJ, Cox MJ, Boden R et al (2007) Stable-isotope probing implicates *Methylophaga* spp and novel Gammaproteobacteria in marine methanol and methylamine metabolism. ISME J 1:480–491 18043650 10.1038/ismej.2007.65

[37] Fischer PQ, Sánchez-Andrea I, Stams AJM, Villanueva L, Sousa DZ (2021) Anaerobic microbial methanol conversion in marine sediments. Environ Microbiol 23(3):1348–1362 33587796 10.1111/1462-2920.15434 PMC8048578

[38] Elías-Arnanz M (2020) Anaerobic bacteria need their vitamin B12 to digest estrogen. Proc Natl Acad Sci U S A 117:1833–1835 31919281 10.1073/pnas.1921340117 PMC6995009

[39] Allison SD, Martiny JBH (2008) Resistance, resilience, and redundancy in microbial communities. Proc Natl Acad Sci U S A 105(supplement_1):11512–9 18695234 10.1073/pnas.0801925105 PMC2556421

[40] Dai L, Vorselen D, Korolev KS, Gore J (2012) Generic indicators for loss of resilience before a tipping point leading to population collapse. Science 336(6085):1175–1177 22654061 10.1126/science.1219805

[41] Lucas-Elio P, Gómez D, Solano F, Sánchez-Amat A (2006) The antimicrobial activity of Marinocine, synthesized by Marinomonas mediterranea, is due to hydrogen peroxide generated by its lysine oxidase activity. J Bacteriol 188:2493–2501 16547036 10.1128/JB.188.7.2493-2501.2006 PMC1428416

[42] Santos AL, Johnson DB (2022) Comparison of different small molecular weight alcohols for sustaining sulfidogenic bioreactors maintained at moderately low pH. Front Bioeng Biotechnol. 10:937987. 10.3389/fbioe.2022.937987. PMID: 36032724; PMCID: PMC9402942. 36032724 10.3389/fbioe.2022.937987 PMC9402942

