# Supplementary material for: Cascade Responses of Microbial Communities To Alcohols and Organic Acids in a Marine Microcosm Experiment
Source: Curr Microbiol. 2025 Nov 13;83(1):21. doi: 10.1007/s00284-025-04581-8 (PMC12615521; doi:10.1007/s00284-025-04581-8)
Supplement: Supplementary file 1 — Supplementary Material 1 [file 284_2025_4581_MOESM1_ESM.docx]

**SUPPLEMENTARY INFORMATION**

**Suppl. Table 1. Functional assignment of zOTUs for 0.5% DOC**

| Function | Cluster I | Cluster II |
| --- | --- | --- |
| methanol_oxidation | 1 | 2 |
| sulfate_respiration | 0 | 1 |
| sulfur_respiration | 1 | 3 |
| dark_sulfite_oxidation | 0 | 1 |
| sulfite_respiration | 0 | 2 |
| thiosulfate_respiration | 0 | 2 |
| arsenate_detoxification | 0 | 1 |
| arsenate_respiration | 0 | 2 |
| nitrate_denitrification | 1 | 3 |
| nitrite_denitrification | 0 | 1 |
| nitrous_oxide_denitrification | 0 | 1 |
| dark_hydrogen_oxidation | 0 | 3 |
| nitrogen_fixation | 1 | 2 |
| nitrate_ammonification | 0 | 2 |
| nitrite_ammonification | 0 | 2 |
| cellulolysis | 0 | 1 |
| xylanolysis | 0 | 1 |
| dark_sulfide_oxidation | 1 | 4 |
| dark_sulfur_oxidation | 0 | 2 |
| dark_thiosulfate_oxidation | 0 | 2 |
| dark_oxidation_of_sulfur_compounds | 0 | 1 |
| manganese_respiration | 0 | 2 |
| fermentation | 1 | 4 |
| aerobic_chemoheterotrophy | 2 | 6 |
| human_pathogens_gastroenteritis | 1 | 1 |
| human_pathogens_diarrhea | 0 | 1 |
| human_pathogens_all | 1 | 2 |
| fish_parasites | 0 | 1 |
| human_gut | 0 | 1 |
| animal_parasites_or_symbionts | 1 | 2 |
| aromatic_hydrocarbon_degradation | 0 | 2 |
| dark_iron_oxidation | 0 | 1 |
| iron_respiration | 1 | 3 |
| nitrate_respiration | 0 | 3 |
| nitrate_reduction | 3 | 5 |
| fumarate_respiration | 1 | 4 |
| predatory_or_exoparasitic | 0 | 1 |
| nonphotosynthetic_cyanobacteria | 0 | 1 |
| anoxygenic_photoautotrophy_S_oxidizing | 0 | 1 |
| photoheterotrophy | 0 | 1 |
| plastic_degradation | 0 | 1 |
| ureolysis | 1 | 3 |
| chemoheterotrophy | 0 | 1 |

**Suppl. Table 2. Functional assignment of zOTUs for 0.05% DOC**

| **Metaboism** | **Cluser I** | **Cluser II** | **Cluser III** |
| --- | --- | --- | --- |
| nitrate_reduction | 3 | 2 | 3 |
| aerobic_chemoheterotrophy | 2 | 1 | 3 |
| nitrate_denitrification | 1 | 1 | 2 |
| nitrogen_fixation | 1 | 1 | 1 |
| dark_sulfide_oxidation | 1 | 1 | 4 |
| human_pathogens_gastroenteritis | 1 | 1 | 1 |
| human_pathogens_all | 1 | 1 | 1 |
| animal_parasites_or_symbionts | 1 | 1 | 1 |
| fumarate_respiration | 1 | 2 | 3 |
| ureolysis | 1 | 1 | 1 |
| methanol_oxidation | 0 | 0 | 1 |
| sulfur_respiration | 0 | 1 | 3 |
| sulfite_respiration | 0 | 0 | 1 |
| thiosulfate_respiration | 0 | 0 | 2 |
| arsenate_respiration | 0 | 0 | 1 |
| dark_hydrogen_oxidation | 0 | 0 | 3 |
| nitrate_ammonification | 0 | 0 | 1 |
| nitrite_ammonification | 0 | 0 | 1 |
| dark_sulfur_oxidation | 0 | 0 | 2 |
| dark_thiosulfate_oxidation | 0 | 0 | 2 |
| dark_oxidation_of_sulfur_compounds | 0 | 0 | 1 |
| manganese_respiration | 0 | 1 | 2 |
| fermentation | 0 | 1 | 2 |
| iron_respiration | 0 | 1 | 2 |
| nitrate_respiration | 0 | 0 | 2 |

**Suppl. Table 3. Curve fitting for the redox response**

| **Place** | **Org** | **percent** | **square** | **linear** | **const** |
| --- | --- | --- | --- | --- | --- |
| Tromsø seafarm | methanol | 0.50 | 6.47 | -79.16 | -148.23 |
| Tromsø seafarm | methanol | 0.05 | 9.13 | -105.27 | -116.43 |
| Tromsø seafarm | ethanol | 0.50 | 3.85 | -56.01 | -232.06 |
| Tromsø seafarm | ethanol | 0.05 | 9.22 | -110.25 | -150.99 |
| Tromsø seafarm | acetic acid | 0.50 | 1.66 | -49.59 | -61.92 |
| Tromsø seafarm | acetic acid | 0.05 | 9.88 | -116.62 | -105.97 |
| Tromsø seafarm | succinic acid | 0.50 | 5.43 | -88.56 | -10.07 |
| Tromsø seafarm | succinic acid | 0.05 | 6.39 | -81.89 | -139.18 |
| Tromsø seafarm | control |  | 3.45 | -39.38 | -133.66 |
| Tromsø refrence | methanol | 0.50 | 3.02 | -64.71 | 6.31 |
| Tromsø refrence | methanol | 0.05 | 6.18 | -87.40 | 12.30 |
| Tromsø refrence | ethanol | 0.50 | 9.14 | -115.85 | -55.29 |
| Tromsø refrence | ethanol | 0.05 | 8.84 | -115.52 | -36.58 |
| Tromsø refrence | acetic acid | 0.50 | 2.84 | -67.94 | 82.60 |
| Tromsø refrence | acetic acid | 0.05 | 9.49 | -133.68 | 72.21 |
| Tromsø refrence | succinic acid | 0.50 | 4.26 | -84.20 | 140.14 |
| Tromsø refrence | succinic acid | 0.05 | 7.72 | -111.47 | 15.48 |
| Tromsø refrence | control |  | 1.88 | -42.36 | -39.77 |
| Oslo fjord | methanol | 0.50 | 3.44 | -43.33 | 61.51 |
| Oslo fjord | methanol | 0.05 | 3.12 | -43.05 | 71.75 |
| Oslo fjord | ethanol | 0.50 | 3.75 | -84.25 | 99.26 |
| Oslo fjord | ethanol | 0.05 | 5.26 | -101.15 | 134.80 |
| Oslo fjord | acetic acid | 0.50 | 1.01 | -10.50 | 193.21 |
| Oslo fjord | acetic acid | 0.05 | 1.41 | -65.81 | 158.49 |
| Oslo fjord | succinic acid | 0.50 | -0.45 | -4.41 | 140.18 |
| Oslo fjord | succinic acid | 0.05 | -1.61 | -28.73 | 87.86 |
| Oslo fjord | control |  | -4.76 | 47.72 | -17.76 |

**Suppl. Table 4. Curve fitting for pH response**

| **Place** | **Org** | **percent** | **square** | **linear** | **const** |
| --- | --- | --- | --- | --- | --- |
| Tromsø seafarn | methanol | 0.50 | 0.01 | -0.12 | 7.74 |
| Tromsø seafarn | methanol | 0.05 | 0.02 | -0.20 | 7.90 |
| Tromsø seafarn | ethanol | 0.50 | 0.03 | -0.29 | 7.86 |
| Tromsø seafarn | ethanol | 0.05 | 0.03 | -0.27 | 7.73 |
| Tromsø seafarn | acetic acid | 0.50 | -0.01 | 0.36 | 4.73 |
| Tromsø seafarn | acetic acid | 0.05 | -0.05 | 0.67 | 5.66 |
| Tromsø seafarn | succinic acid | 0.50 | -0.03 | 0.56 | 4.35 |
| Tromsø seafarn | succinic acid | 0.05 | -0.03 | 0.44 | 6.16 |
| Tromsø seafarn | control |  | 0.01 | -0.08 | 7.61 |
| Tromsø reference | methanol | 0.50 | 0.02 | -0.20 | 7.95 |
| Tromsø reference | methanol | 0.05 | 0.02 | -0.21 | 7.97 |
| Tromsø reference | ethanol | 0.50 | 0.01 | -0.20 | 7.90 |
| Tromsø reference | ethanol | 0.05 | 0.02 | -0.21 | 7.93 |
| Tromsø reference | acetic acid | 0.50 | -0.02 | 0.43 | 4.73 |
| Tromsø reference | acetic acid | 0.05 | -0.02 | 0.36 | 6.32 |
| Tromsø reference | succinic acid | 0.50 | 0.01 | 0.21 | 4.72 |
| Tromsø reference | succinic acid | 0.05 | -0.04 | 0.52 | 6.01 |
| Tromsø reference | control |  | 0.00 | -0.04 | 7.66 |
| Oslo fjord | methanol | 0.50 | 0.00 | -0.03 | 7.67 |
| Oslo fjord | methanol | 0.05 | 0.01 | -0.08 | 7.70 |
| Oslo fjord | ethanol | 0.50 | 0.01 | -0.15 | 7.85 |
| Oslo fjord | ethanol | 0.05 | 0.02 | -0.20 | 7.88 |
| Oslo fjord | acetic acid | 0.50 | -0.01 | 0.16 | 3.74 |
| Oslo fjord | acetic acid | 0.05 | -0.02 | 0.49 | 5.04 |
| Oslo fjord | succinic acid | 0.50 | -0.01 | 0.17 | 3.68 |
| Oslo fjord | succinic acid | 0.05 | -0.05 | 0.78 | 4.91 |
| Oslo fjord | control |  | 0.00 | 0.05 | 7.60 |
